# Introducing the Topical Collection: ‘Climate change communication and the IPCC’

**DOI:** 10.1007/s10584-021-03253-3

**Published:** 2021-12-02

**Authors:** Saffron O’Neill, Roz Pidcock

**Affiliations:** 1 College of Life and Environmental Sciences, University of Exeter, Amory Building, Rennes Drive, Exeter, EX4 4RJ UK; 2 Independent consultant, Quercus Group, Chichester, West Sussex UK

The Intergovernmental Panel on Climate Change (IPCC) is widely regarded as the most important and authoritative source on climate change, its impacts and how to tackle the rising emissions that drive it. With this authority comes a responsibility to ensure the information is communicated effectively to policymakers, citizens and those who rely on the information for their lives and livelihoods. How the IPCC communicates the information in its reports via its official materials (e.g. Summary for Policymakers, presentations, FAQs), through different channels (e.g. interaction with journalists, social media, outreach events in different countries) and to its main audience of policymakers as well as others (including media, business, NGOs, education) has been the subject of intense analysis in the past. How different types of evidence are included in IPCC reports, particularly indigenous and local knowledge, is also a rich vein of discussion. Similarly, the representativeness of the leadership, staff and author teams in terms of gender, geographical balance and diversity of expert perspectives is key to ensuring all voices are heard and all relevant evidence is considered.

The IPCC has a long history of asking the research and global communications communities for input to its evolving communications strategy. In 2016, the IPCC convened an Expert Meeting on Communication, which led to a number of recommendations to enhance IPCC communications activities, strategy and capacity (IPCC 2016; see also Lynn and Peeva, this issue). With the Sixth Assessment Report (AR6) cycle nearing completion,^1^ now is an important moment to, once again, take stock of the evolving IPCC communications strategy, a time to critically reflect on successes, challenges, lessons learned and best practice for future reports. We also intend for this Topical Collection (TC) to speak to other institutions seeking to further their climate science engagement efforts at global, national, regional and local scales.

## Vision for the TC

This TC is Guest Edited by Saffron O’Neill and Roz Pidcock, an academic-practitioner team. It came about through our mutual interest and ongoing commitment to theoretical and empirical rigour in climate communication research, alongside a commitment towards seeking to implement and learn from climate communications best practice. O’Neill has researched climate communication for over a decade, including bringing together academics and communications practitioners for a Focus Issue titled: *IPCC and media coverage of climate reports* in the journal *Nature Climate Change* (2015). Pidcock is a writer, editor and climate change engagement specialist and was Head of Communications for Working Group I of the IPCC for the publication of the Special Report on 1.5C.

The TC Call for Papers was advertised widely, aiming to bring together a diversity of voices as part of efforts to open up the global-north dominated literature (c.f. Moser 2016) and to include a range of perspectives from early career to established voices. TC academic authors represent climate communication expertise from across a broad range of disciplines including Agriculture, Anthropology, Climate Science, Environmental Studies, Geography, Media and Journalism, Psychology and Sociology. Considering the current trend of a significant fall in journal article authorship by women during the COVID-19 pandemic (Viglione 2020), we are pleased to report that over half of the contributions are lead-authored by women. The progress of this TC has been considerably delayed by the pandemic, with the task of finding available peer-reviewers with appropriate expertise especially time-consuming. In this context, we wish to convey our sincere thanks to the 50 + anonymous reviewers who contributed to the TC. Your careful work represents an unseen but vital part of this collection.

This TC brings together perspectives from academics and communication practitioners, as well as IPCC voices (roles which are not necessarily mutually exclusive). Whilst the TC has been fully independent of the IPCC, there has been significant engagement with the IPCC throughout the TC, resulting in a number of contributions lead- or co-authored by IPCC staff. Jonathan Lynn, outgoing Head of Communications and Media Relations at the IPCC Secretariat, has lead-authored an introduction to IPCC communication history, and Thelma Krug, IPCC Vice-Chair, has reflected on the key themes of the TC in a concluding synthesis commentary. Whilst contributors to our Topical Collection make recommendations for the attention of ‘the IPCC’ as a whole entity, we wish to acknowledge the many moving parts that, in reality, make up the complex IPCC machine, not all of whom it has been possible to represent in this Topical Collection. That long list includes the IPCC leadership, secretariat, technical support units, authors (coordinating, lead and contributing), government and expert reviewers, review editors, chapter scientists, contractors and many more, alongside the IPCC’s 195 Member Countries.

## Themes and contributions

Lynn and Peeva begin the Topical Collection by laying out the history and innovations in IPCC communication strategy. It is evidence from Fig. 1 of their paper (Lynn and Peeva 2021) that there has been tremendous progress on communication as a key issue for the IPCC. Yet as opportunities have been taken and innovations progressed, it is also evident that there are significant communication challenges. Communication clearly lays at the heart of the IPCC: as Lynn and Peeva quote, and worth repeating here, this ‘brutal question’ was posed by IPCC Chair Hoseung Lee at a Side Event at COP21 in Paris, in the lead up to the Oslo Expert Meeting on Communication in 2016: ‘what use are IPCC reports if many of the intended users cannot understand them, do not know where to find them, or cannot use them in their own work?’ (Lee 2015:1).

The TC speaks to these challenges posed by Lee. Some of the contributions suggest concrete and fairly straightforwardly actionable proposals, which fit within the bounds of IPCC communication strategy to date—indeed, some investigate recent changes in IPCC communication strategy (Morelli et al., Pidcock et al.; see also Pathak et al., Mcloughlin, Connors et al.); though others do highlight significant challenges ahead, such as the role of social media for the IPCC (e.g. Sanford et al.). Alongside these contributions, which fit more or less neatly into existing structures, other papers present a more fundamental challenge to IPCC engagement and communication. These papers ask questions such as: whose knowledge is valued, and to what end? (e.g. see Asayama, Chakraborty and Sherpa, Dudman and de Wit, Hermansen et al.). For papers that fit within (or challenge) the status quo though, one key piece of information must be borne in mind. Any discussion of IPCC communication strategy needs to first be centred in the IPCC’s mandate of providing ‘policy relevant but not-prescriptive’ assessments (see Schipper et al. 2021). It is in how papers speak to this mandate that dictates whether they can be more or less easily accommodated within the existing institution that is the IPCC. That said, our view as Guest Editors is that this mandate should serve as a prompt for the IPCC to be responsive to the needs and expectations of its audiences, rather than act as a refuge for intransigence. Indeed, regularly reflecting on its own definition of ‘policy relevance’ could be fruitful for the IPCC to ensure it stays societally relevant.

The first four contributions are from authors deeply connected to, or embedded in, the IPCC communications landscape. The essay from Morelli et al. describes a process of co-design for data visualisations that has been refined through the AR6 drafting process. They lay out three crucial elements to a successful co-design process: practical tools and a flexible method, social science expertise to understand the needs of users and the importance of trust and leadership. Morelli et al. point towards an evolving design culture within the IPCC, one which recognises the value of a visual story, whilst it retains scientific integrity. Led by an author at WGI’s Technical Support Unit (TSU), Connors et al. also use their essay to argue for co-design but in this case for the development of the IPCC’s Frequently Asked Questions (FAQs). As the only mandatory part of the Assessment Reports (AR) targeting a non-expert audience, the FAQs represent an important distillation of IPCC findings. Connors et al. make a number of recommendations for FAQ co-development in subsequent ARs, including a sharing of responsibility for FAQs between communications experts and scientists, as well as common FAQ guidelines across the WGs (which have so far developed separately). In a third contribution embedded within existing IPCC communications strategy, Pidcock et al. call for better integration of the theory of effective public engagement with climate science. In their article, they lay out findings from a global survey which gathered practical examples of outreach and engagement by WGI authors. These are juxtaposed against key principles of effective engagement, as expressed in the Communications Handbook for IPCC authors, in order to highlight and find opportunities to promote best practice. Last, Pathak et al. bring a welcome perspective from a Global South context. In their essay, they discuss a wealth of in-person outreach activities carried out across India. Although these activities varied considerably in scale, audience and topic, some commonalities emerged. Pathak and her co-authors raise the considerable challenges of communicating to diverse audiences, of limited resources and of translation into local languages, with several suggestions for addressing these.

The importance of different communication devices is highlighted in an article led by Bruine de Bruin and in two essays: one from Mcloughlin and another from Bloomfield and Manktelow. Bruine de Bruin and colleagues present evidence of the (mis)understanding of key terminology from the climate domain. Using a qualitative interview methodology, speaking to US residents, common themes were identified. All those involved in climate communications should heed their findings that even terms like ‘mitigation’ and ‘carbon neutral’ were perceived as difficult to understand. Indeed, even when terms were considered easier to understand (like ‘adaptation’), participants struggled to make the connection between the common usage of the word and its potential definition in a climate context.

Mcloughlin’s essay considers the role of efficacy (‘beliefs about personal or collective capacity to respond and the effectiveness of responses’) in IPCC communications. He lays out the roles of three different types of efficacy (self, response, collective), providing examples of where these could be invoked in a series of examples. Mcloughlin recognises the potential limits to nurturing efficacy in IPCC communications and makes suggestions of where other allied organisations may be able to make more targeted appeals to efficacy. Bloomfield and Manktelow argue for a set of more engaging Summary for Policymakers (SPMs) by considering the role of storytelling and by incorporating narrative features. They evaluate the AR5 SPM for storytelling opportunities, outlining how communications theory could usefully intervene to bring about narrative changes to identify characters, settings and morals and bring in engaging comparisons and analogies.

The opportunities and challenges of social media are picked up in a number of contributions. Eide et al. draw from interviews with more than 30 international youth climate activists to show how IPCC science plays a central role in their activities. Notably, they highlight the networked communication landscape in which these voices become prominent and the possibilities for interaction that these online spaces create. The opportunities for engagement online are also picked up in Sanford et al., where they note a corpus of over 27,000 tweets in 41 languages during August 2019, around the launch of the Special Report on Climate Change and Land. However, Sanford and colleagues are also clear about how the IPCC needs to respond more effectively to distortions of report contents on social media platforms, to deepen their understanding of online climate communication and to more fully come to terms with how the digital landscape impacts the wider climate communications environment.

The last four papers all challenge the workings of the IPCC, and hence how and why it communicates, in more fundamental ways than the papers outlined previously. Hermansen et al. start from the concept of ‘policy relevance […] the ‘raison d’être’ of the IPCC. They present an analysis of IPCC policy relevance across differing scales from global, to regional, to national. They conclude that the IPCC should acknowledge policy relevance not only in process, but also in terms of policy relevance as outcome. They present three recommendations for how the IPCC can work constructively to pursue policy relevance. Asayama also speaks to conceptualisations of the science-policy relationship. Whilst he maintains that in principle, the IPCC has remained policy-neutral; he contends that in practice, the IPCC has acted as a powerful discursive agent, enabling a discourse of scarcity to gain prominence. Asayama argues for an emancipatory discourse instead, calling for more meaningful engagement with the interpretive disciplines within social sciences and humanities in order to progress this aim. Similarly, Dudman and de Wit also critique ‘whose knowledge counts?’ at the IPCC table. They suggest a way forward for the IPCC to engage—and more particularly, to listen—more effectively to diverse forms of knowledge and to recognise the until-now privileging of scientific knowledge over other types or domains of knowledge. Last, Chakraborty and Sherpa eloquently describe their personal experiences with local communities in the Himalayas, juxtaposing it with their experiences in the IPCC knowledge production process. Despite considerable efforts by the IPCC to address these, they call out the IPCC’s still-existing biases: towards the Global North, voices of men, natural science disciplinary representation and western science cosmology over indigenous knowledges. Their experiences documented in the essay highlights marginalised narratives of climate-society relationships that challenge the existing understanding of the science-policy relationship, as well as highlighting issues of equity and justice.

It is fitting that the TC is bookended with a contribution from IPCC Vice Chair Thelma Krug, reflecting on the challenges and opportunities posed by the TC contributions, speaking to the question: ‘where now for the IPCC on climate communication’? Again, we should note that this TC project was independent of the IPCC: but we are delighted that the contributions herein have contributed to, and sparked discussion at, the highest levels of the IPCC. We sincerely thank her for this critical engagement with the essays and articles, and we hope that this forms part of a continuation of learning for us all on climate change communications.

## Looking forward

The essays and articles in this TC raise many considerations for the IPCC to reflect on in terms of its evolving communications strategy. Drawing from these contributions, and our own perspectives, we offer the following overarching suggestions in our roles as Guest Editors, as the IPCC begins to look beyond AR6:

- Evolving the existing communication strategy (and separate implementation strategy) into a comprehensive IPCC Engagement Strategy. This would signal a shift from one-directional delivery towards two-way dialogue and be the basis for nurturing a more participatory approach to ensuring the report is relevant to the needs of a diverse, global audience. As part of this, the IPCC could explore feasible avenues for interacting meaningfully with key audiences at all stages of the report process, from incorporating diverse forms of knowledge to disseminating the findings.
- Revamping the existing review process to include more opportunities for key stakeholders to contribute to and/or ‘road test’ specific elements of the report and associated communication materials upstream of their production. For example, the IPCC could consider if there is flexibility within its working practices to incorporate principles of representative deliberation, such as a Citizens Panel, whilst adhering to IPCC guiding principles. These sorts of deliberative processes are increasingly used to tackle complex issues, convening diverse groups of people to learn and contribute to public issues (OECD 2020). In an IPCC context, such a panel could be used (as a minimum) to seek feedback on how relatable various concepts, narratives and language are perceived to be and to test how evolving visuals are interpreted, for example.
- Whilst the addition of communications specialists in the Working Groups represents a significant and much-needed step forward, a more sophisticated approach to what it means to ‘embed’ communications capacity with the IPCC is needed. Re-conceptualising IPCC communications capacity as a ‘distributed system’ rather than operating as separate entities housed within the Working Groups and Secretariat would allow a more efficient operation, both internally and externally.

These three points are proposed as potential fruitful lines of enquiry for the IPCC and as prompts for further discussion. We thank all the Topical Collection authors and IPCC staff for their engagement to date; and we look forward to these conversations opening up further in future.

## Data Availability

Not applicable.

## Footnotes

1 The WGI Report was published in August 2021. The WGII Report is due for release in February 2022, WGIII in March 2022, and the Synthesis Report is due in October 2022
